# Management of chyle leakage after general thoracic surgery: Impact of thoracic duct embolization

**DOI:** 10.1111/1759-7714.13914

**Published:** 2021-03-30

**Authors:** Yeong Jeong Jeon, Jong Ho Cho, Dongho Hyun, Sumin Shin, Hong Kwan Kim, Yong Soo Choi, Jhingook Kim, Jae Ill Zo, Young Mog Shim

**Affiliations:** ^1^ Department of Thoracic and Cardiovascular Surgery, Samsung Medical Center Sungkyunkwan University School of Medicine Seoul Korea; ^2^ Department of Radiology, Samsung Medical Center Sungkyunkwan University School of Medicine Seoul Korea

**Keywords:** chyle leakage, thoracic duct embolization, thoracic duct ligation

## Abstract

**Background:**

The aim of this study was to investigate the impact of thoracic duct embolization (TDE) on the management of postoperative chyle leakage.

**Methods:**

We retrospectively reviewed the electronic medical record database of 4171 patients who underwent curative resection for lung or esophageal cancer between January 2015 and June 2017. We classified the period before the introduction of TDE as the first period and the period after the introduction of TDE as the second period.

**Results:**

A total of 105 patients who developed chyle leakage after surgery were included. In the first period, 49 patients who underwent lung surgery developed chylothorax. Of those, two patients (4.1%) underwent surgical ligation of the thoracic duct (TD). Of eight patients with chyle leakage after esophagectomy, four patients (50%) underwent TD ligation. In the second period, 30 patients developed postoperative chyle leakage after pulmonary resection. Only one (3.3%) of them required surgical ligation. Of eight patients with chyle leakage after esophagectomy, only two (11.1%) patients underwent TD ligation. Five patients (16.7%) received TDE after lung surgery and five patients (27.7%) after esophageal surgery. Also, in the second period, the hospital stay of patients who underwent lung cancer surgery was shorter than the first period (12.6 ± 4.6 days vs. 16.3 ± 9.7 days; *p* = 0.026).

**Conclusions:**

TDE is an effective method for the management of chyle leakage and might help to avoid invasive surgery.

## Graphical abstract

After the introduction of thoracic duct embolization (TDE), only three of 48 patients underwent surgical ligation of thoracic duct. The length of hospital stay of patients who underwent lung cancer surgery was shorter than that before the introduction of TDE. This study demonstrated that TDE is an effective method for the management of postoperative chyle leakage and can help avoid invasive surgery in this group of patients.

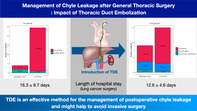

## INTRODUCTION

Postoperative chyle leakage is troublesome and sometimes difficult to control. The incidence rate of chyle leakage after general thoracic surgery has been reported to range from 0.4% to 3.9%.^1^, ^2^, ^3^ General thoracic surgery is performed in the field close to the thoracic duct (TD), and therefore may potentially disrupt the main TD or its tributaries. In patients who undergo pulmonary resection, TD injury usually occurs during mediastinal lymph node dissection.^4^ In patients who undergo esophagectomy, extended lymphadenectomy is associated with damage of the TD and the lymphatic collateral system.^5^

Chyle leakage is a serious complication with a mortality rate ranging from 0% to 50%.^3^, ^6^, ^7^ Persistent chyle leakage can cause loss of fluids, lymphocytes, and albumin. This may lead to malnutrition and immune dysfunction, resulting in infection‐related complications such as pneumonia and sepsis.^8^

Traditionally, conservative management has included no enteral feeding and total parenteral nutrition or a low‐fat diet. High‐output chylothorax usually required intervention, including TD ligation or embolization. Percutaneous treatment of chylothorax was introduced in 1998.^9^ Thoracic duct embolization (TDE) has been suggested over the past 20 years as a minimally invasive alternative to surgical ligation. The aim of this study was to investigate the effectiveness of TDE for the management of postoperative chyle leakage comparing outcome before and after its introduction.

## METHODS

### Patients

We retrospectively reviewed the electronic medical record database between January 2015 and June 2017. We have recorded postoperative complications based on Clavien‐Dindo classification since 2015, and TDE has been performed at our institution since May 2016. Therefore, we classified the period before the introduction of TDE (January 2015–April 2016) as the first period and the period after the introduction of TDE (May 2016–June 2017) as the second period. The incidence of chyle leakage and subsequent therapeutic management were reviewed. This study received ethical approval from the institutional review board and consent of patients was waived.

Chyle leakage was suspected if the drainage was milky or a cloudy color, or if the amount of drainage rapidly increased after feeding. Triglyceride levels were measured in patients suspected of having chyle leakage to confirm the diagnosis. Chyle leakage was defined as a milky drainage with a triglyceride level greater than 110 mg/dl.

### Management of chyle leakage

In the first period, all patients who had chyle leakage were treated primarily with conservative management. Conservative management included no enteral feeding and total parenteral nutrition or a low‐fat diet. If chyle leakage did not decrease with conservative management, other interventions were attempted, including pleurodesis and administration of somatostatin. If the drainage continued and exceeded more than 1000 ml/day, even after nonsurgical intervention, surgical ligation of the TD was performed based on the judgment of the surgeon. In the second period, all patients with chyle leakage who failed to improve with conservative management attempted TDE, except when additional surgery was required for other reasons.

### Procedure

We usually use magnetic resonance lymphangiography to visualize the TD and cisterna chyli at the embolization planning stage. The inguinal nodes were accessed percutaneously under direct ultrasound guidance. After lipiodol was injected, the lymphatic channels of interest, including the cisterna and TD, were opacified. Either the cisterna chyli or TD was accessed via a transabdominal approach, and a microcatheter was then advanced over the guidewire. Once the leakage was identified with angiography using an iodine contrast agent, the microcatheter was advanced past the leakage, and microcoils or diluted glue were used to embolize the branch of TD or TD itself. If the TD was challenging to approach, needle interruption of the TD was performed. The definition of failure for TDE was technical failure including unsuccessful catheterization of the thoracic duct and subsequent embolization

### Statistical analysis

We compared the patients in the first period with the patients in the second period using a Student's *t*‐test or the Mann–Whitney test, depending on the normality of distribution, for continuous variables, and the Pearson's chi‐square and Fisher's exact tests for categorical variables, when appropriate. A *p‐*value of less than 0.05 was considered statistically significant. All analyses were performed with SPSS 25 (SPSS Inc).

## RESULTS

A total of 4171 patients underwent curative surgery for lung or esophageal cancer. Of these, 105 patients who developed chyle leakage after surgery were included in the current study. The median age of the patients was 63 years (age range, 33–85 years). The frequency of chyle leakage was much higher in right‐sided operations than left‐sided operations (84.8% and 13.3%, respectively). Patients underwent TDE or surgical ligation at a median of five days (range, 3–9 days) after diagnosis of chyle leakage. Interval between diagnosis of chyle leakage and surgical ligation was a median of nine days (range, 3–9 days), whereas time to TDE was a median of 4.5 days (range 3.25–8.25 days). The first period had more patients who underwent surgery for lung cancer than the second period (86% and 62.5%, respectively). The details are described in Table 1.

**TABLE 1:** Baseline characteristics of patients who developed chyle leakage after surgery

		First period(*n* = 57)	Second period(*n* = 48)	*p*‐value
Age	(Median, range)	62 (33–85)	65 (42–80)	0.318
Sex	Female	23 (40.3%)	12 (25%)	
	Male	34 (59.7%)	36 (76%)	0.146
Site	Right	46 (82.1%)	43 (91.5%)	0.276
	Left	10 (17.9%)	4 (8.5%)	
Approach	Open thoracotomy	23 (40.4%)	29 (60.4%)	0.064
	Minimally invasive surgery	34 (59.6%)	19 (39.6%)	
Surgery	Lung cancer	49 (86%)	30 (62.5%)	0.011
	Esophageal cancer	8 (14%)	18 (37.5%)	
Lymph node dissection	Yes	56 (98.3%)	48 (100%)	1.000
	No	1 (1.8%)	0	
Treatment	Low‐fat diet	13 (22.8%)	9 (18.8%)	
	NPO ± low‐fat diet	36 (63.2%)	26 (54.2%)	
	Surgical ligation	6 (10.5%)	3 (6.3%)	
	Thoracic duct embolization	0	10 (23.8%)	
Hospital stay	(days, mean ± SD)	19.1 ± 4.5	18 ± 13	0.346
Time to oral feeding	(days, mean ± SD)	5.8 ± 7	7.3 ± 6.4	0.253
Peak drainage amount	(cc/day, mean ± SD)	683.3 ± 489.4	784.1 ± 653.5	0.369

Abbreviations: NPO, nil per os (nothing by mouth); SD, standard deviation.

In the first period, 57 out of 2176 patients (2.6%) had chyle leakage, 49 after lung cancer surgery and eight after esophageal cancer surgery. Of the 49 patients who developed chylothorax after surgery for lung cancer, two patients (4.1%) underwent surgical ligation of the TD, whereas 45 (91.9%) patients were treated with conservative management. In addition, in eight patients with chyle leakage after esophagectomy, four patients (50%) underwent TD ligation, whereas three patients (62.5%) were treated conservatively.

**FIGURE 1:**
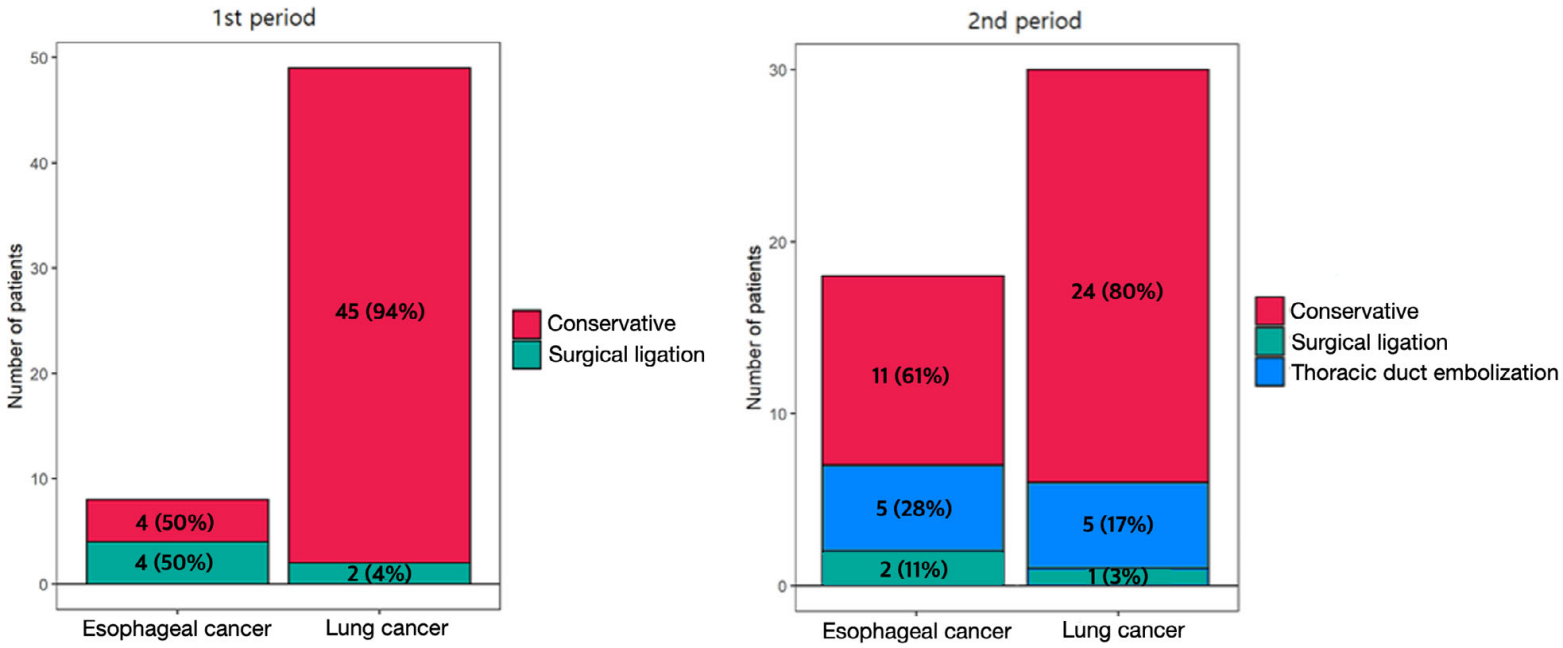
Management of patients with chyle leakage

On the other hand, in the second period, 48 out of 1995 patients (2.4%) developed postoperative chyle leakage (30 after pulmonary resection and 18 after esophagectomy), but only one (3.3%) of the patients who underwent pulmonary resection and two (11.1%) who underwent esopahgectomy required surgical ligation. Compared to the first period, the number of patients who underwent surgical ligation of TD was reduced in the second period. Five patients (16.7%) received TDE after lung surgery and five patients (27.7%) after esophageal surgery (Figure 1).

In the second period, the length of hospital stay for patients who underwent lung cancer surgery was shorter than in the first period (12.6 ± 4.6 days vs. 16.3 ± 9.7 days; *p* = 0.026). However, the hospital stay for patients who underwent esophageal cancer surgery was not different between before and after the introduction of TDE (35.9 ± 25.9 days and 26.8 ± 17.3 days, respectively). Time to oral feeding and peak drainage amount were not different between the two periods. Treatment and outcomes of patients according to the surgery are summarized in Tables 2 and 3.

**TABLE 2:** Treatment and outcomes of patients who underwent lung cancer surgery

		First period(*n* = 49)	Second period(*n* = 30)	*p*‐value
Treatment	Low‐fat diet	12 (24.5%)	6 (20%)	
	NPO ± low‐fat diet	33 (67.4%)	18 (60%)	
	Surgical ligation	2 (4.1%)	1 (3.3%)	
	Thoracic duct embolization	0	5 (16.7%)	
Hospital stay	(days, mean ± SD)	16.3 ± 9.7	12.6 ± 4.6	0.026
Time to oral feeding	(days, mean ± SD)	5 ± 4.5	4.8 ± 3.1	0.837
Peak drainage amount	(cc/day, mean ± SD)	565.2 ± 303.3	575.8 ± 454.9	0.901

Abbreviations: NPO, nil per os (nothing by mouth); SD, standard deviation.

**TABLE 3:** Treatment and outcomes of patients who underwent esophageal cancer surgery

		First period(*n* = 8)	Second period(*n* = 18)	*p*‐value
Treatment	Low‐fat diet	1 (12.5%)	3 (16.7%)	
	NPO ± low‐fat diet	3 (37.5%)	8 (44.4%)	
	Surgical ligation	4 (50%)	2 (11.1%)	
	Thoracic duct embolization	0	5 (27.8%)	
Hospital stay	(days, mean ± SD)	35.9 ± 25.9	26.8 ± 17.3	0.302
Time to oral feeding	(days, mean ± SD)	17.3 ± 9.1	12.8 ± 6.1	0.166
Peak drainage amount	(cc/day, mean ± SD)	1406.8 ± 768.1	1131.2 ± 789.7	0.416

Abbreviations: NPO, nil per os (nothing by mouth); SD, standard deviation.

**FIGURE 2:**
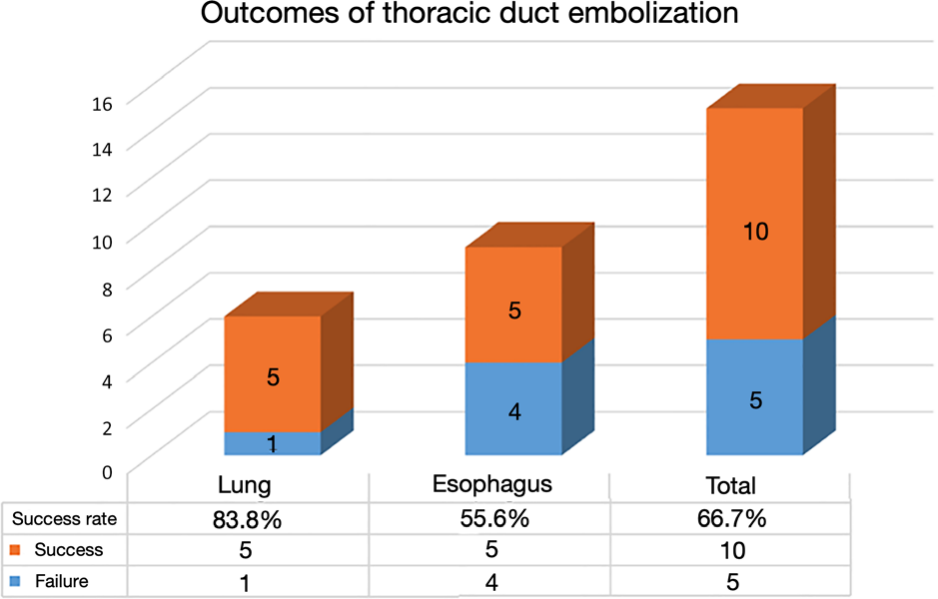
Outcomes of thoracic duct embolization

The success rate for TDE in patients who underwent lung cancer surgery was 83.3%, whereas the success rate in patients who underwent esophageal cancer surgery was 55.6%. This result is illustrated in Figure 2.

## DISCUSSION

Postoperative chyle leakage is a well‐known complication after thoracic surgery. We found that the incidence of chyle leakage was 2.2% in patients who underwent lung cancer surgery and 4.3% in patients who underwent esophageal cancer surgery. This is in line with previously reported studies.^3^, ^5^, ^10^, ^11^

Earlier studies have reported successful outcomes with conservative management.^12^, ^13^, ^14^ Park et al. demonstrated that 67 patients with chylothorax after lung cancer surgery were successfully treated by no enteral feeding or a low‐fat diet with or without pleurodesis.^15^ Fujita et al. reported that conservative treatment with octreotide had a successful resolution rate of chylothorax (86%).^16^ Some surgeons have advocated that early surgical management avoided high morbidity in patients with high‐output chyle leakage and reported better outcomes than with conservative treatment.^6^, ^10^, ^17^

Since the introduction of TDE by Cope and Kaiser, TDE has served as an alternative to invasive surgery. Cope and Kaiser reported a success rate of 73.8% for TDE among 42 patients with both traumatic and nontraumatic chylothorax.^18^ Itkin et al. reported outcomes of TDE in 109 patients with chyle leakage, with a success rate of 71%. In cases in which catheterization was successful, the resolution rate of chylothorax was 90%.^19^ In addition, Chen et al. published the long‐term complications of TDE. They analyzed 169 patients who underwent TDE. TDE was technically successful in 115 patients (68%). Among 57 patients who were alive at the time of follow‐up, four patients experienced chronic leg swelling and five patients complained of procedure‐related chronic diarrhea.^20^

In the present study, we demonstrated that TDE was an effective method for the management of chyle leakage. After the introduction of TDE, only three of 48 patients underwent surgical ligation of the thoracic duct. In addition, the length of hospital stay of patients who underwent lung cancer surgery was shorter than that before the introduction of TDE. Prior to the introduction of TDE, if chyle leakage was not resolved even with conservative treatment, surgical ligation under general anesthesia had to be considered. Due to the burden of this option, conservative treatment continued for more than one week. However, after the introduction of TDE, we attempted TDE instead of surgical ligation if high‐output chyle leakage persisted despite conservative treatment. When TDE was technically successful, chyle leakage resolved in all patients and surgical ligation was avoided in these patients. Therefore, in the era of noninvasive TDE, chyle leakage could resolve earlier by performing TDE in the early period of chyle leakage, thus leading to a reduction in hospital stay.

The technical success rate of TDE in patients who underwent esophageal surgery was 55.6%, whereas the success rate in patients who underwent lung surgery was 83.3%. Among five patients who failed TDE, three patients underwent surgical ligation and two patients waited with conservative management. From a technical point of view, TDE for chyle leakage after esophageal cancer is more challenging than TDE after lung cancer. After lung cancer surgery, chyle leakage frequently occurs from branch lymphatic channels at the mediastinal lymph node dissection such as the lower paratracheal nodal station. In this case, TD catheterization via a transabdominal approach is usually possible. If transabdominal access is not available, a retrograde approach via the subclavian vein or direct puncture of the cervical portion of the thoracic duct is an alternative option. On the other hand, during esophageal cancer surgery, the extent of lymph node dissection tends to be wider so that either the TD or even the cisterna chyli or neck lymph nodes can be sacrificed. When those central lymphatic structures are injured, both lymphangiography and transabdominal or retrograde cannulation of the TD can be technically infeasible. Furthermore, lymphatic channels can be injured at paracardial nodes, lymph nodes along the lesser curvature and left gastric artery which are hardly accessible via the conventional lymphangiographic technique. Accordingly, either technical or clinical success of TDE is less likely.

The current study has both weaknesses and strengths. Because of the retrospective nature of this study, there might be a selection bias that could influence treatment outcomes, particularly regarding the period of hospital stay. The current study was conducted at a single tertiary center. To generalize the outcomes, our results need to be validated with a larger and multicenter study. However, our results are worth noting in that the current study demonstrated not only treatment efficacy of TDE, which has been reported in the literature, but also its impact on the management of postoperative chyle leakage by comparing the periods before and after lymphatic intervention..

In conclusion, postoperative chyle leakage is a serious complication and sometimes difficult to control. After the introduction of TDE, the length of hospital stay of patients who underwent lung cancer surgery was shorter than that before the introduction of TDE. TDE is an effective method for the management of postoperative chyle leakage and might help to avoid invasive surgery.

## CONFLICT OF INTEREST

The authors declare no potential conflicts of interest with respect to the research, authorship, and/or publication of this article.
